# Transcriptional Repression of Raf Kinase Inhibitory Protein Gene by Metadherin during Cancer Progression

**DOI:** 10.3390/ijms22063052

**Published:** 2021-03-17

**Authors:** Trang Huyen Lai, Mahmoud Ahmed, Jin Seok Hwang, Sahib Zada, Trang Minh Pham, Omar Elashkar, Deok Ryong Kim

**Affiliations:** Department of Convergence Medical Science and Biochemistry, Institute of Health Sciences, Gyeongsang National University School of Medicine, Jinju 527-27, Korea; Tranghuyen20493@gmail.com (T.H.L.); mahshaaban@gnu.ac.kr (M.A.); cloud8104@naver.com (J.S.H.); s.zada.qau@gmail.com (S.Z.); phamminhtrang010895@gmail.com (T.M.P.); omar.i.elashkar@gmail.com (O.E.)

**Keywords:** RKIP/PEBP1, MTDH/AEG-1, VEZF1, transcription factor, RKIP expression, ChIP assay, promoter assay

## Abstract

Raf kinase inhibitory protein (RKIP), also known as a phosphatidylethanolamine-binding protein 1 (PEBP1), functions as a tumor suppressor and regulates several signaling pathways, including ERK and NF-κB. RKIP is severely downregulated in human malignant cancers, indicating a functional association with cancer metastasis and poor prognosis. The transcription regulation of *RKIP* gene in human cancers is not well understood. In this study, we suggested a possible transcription mechanism for the regulation of RKIP in human cancer cells. We found that Metadherin (MTDH) significantly repressed the transcriptional activity of *RKIP* gene. An analysis of publicly available datasets showed that the knockdown of *MTDH* in breast and endometrial cancer cell lines induced the expression RKIP. In addition, the results obtained from qRT-PCR and ChIP analyses showed that MTDH considerably inhibited RKIP expression. In addition, the RKIP transcript levels in *MTDH*-knockdown or *MTDH*-overexpressing MCF-7 cells were likely correlated to the protein levels, suggesting that MTDH regulates RKIP expression. In conclusion, we suggest that MTDH is a novel factor that controls the RKIP transcription, which is essential for cancer progression.

## 1. Introduction

Raf Kinase Inhibitory Protein (RKIP; also called PEBP1) was first elucidated as a binding protein of Raf1, a key regulator in mitogen-activated protein kinase (MAPK) pathways. RKIP is ubiquitous in cellular structures, including cytoplasm, inner periplasmic membrane, and others [1]. Low expression of RKIP protein has been reported in many human cancers, including metastatic prostate, breast, and colon cancers, hepatocellular carcinoma, melanomas, and insulinomas [2]. Thus, lack of RKIP protein activates the MEK/ERK pathway, consequently promoting cell proliferation, survival, differentiation, and migration during cancer progression [3,4,5]. As a result, RKIP is considered a diagnostic biomarker associated with cancer metastasis and poor prognosis. However, the mechanism responsible for the low expression of RKIP in human cancers is not well understood. One possible mechanism for the low intracellular level of RKIP in human diseases could involve reducing the *RKIP* gene transcription. To test this possibility, we investigated transcription factors and co-factors that directly or indirectly regulate the expression of the *RKIP* gene.

The oncogene Metadherin (MTDH, also known as astrocyte elevated gene 1, AEG1) was first identified as a novel transcript in the primary human fetal astrocytes (PHFAs) infected with human immunodeficiency virus (HIV)-1 and is specifically elevated in astrocytes [6]. MTDH is generally localized in the nucleus and cytoplasm as well as at the plasma membrane and is functionally associated with several oncogenic signaling pathways, such as PI3K/AKT pathway, and transcription factors, such as nuclear factor NF-κB. Many studies show that MTDH is overexpressed in all solid tumors, including breast, prostate, gastric, renal, colorectal, ovarian, and endometrial cancers [7,8,9]. A study suggests that MTDH could modulate gene expression by acting as a co-factor through its nuclear homing domain, as it does not contain any domains necessary for the direct DNA binding [10]. In this study, we examined whether MTDH plays a critical role in regulating RKIP expression as a transcription co-factor. We found that MTDH associates with the *RKIP* promoter and significantly inhibits the expression of RKIP. This suggests that MTDH negatively regulates the transcription of the *RKIP* gene, which could be a target for the development of new cancer therapy.

## 2. Results

### 2.1. MTDH Is Upregulated in Cancer Tissues with Low Frequency Of Genetic Alterations

**Figure 1:**
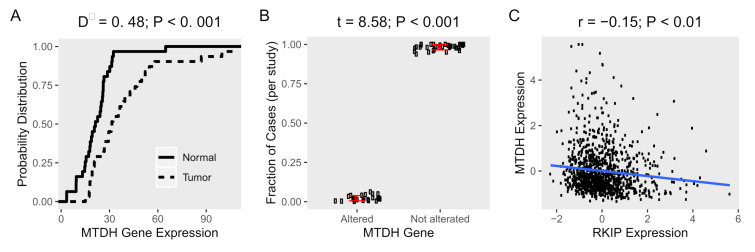
Expression and alterations of *MTDH* gene in cancer tissues. Gene expression data of human samples and matched normal tissues (*n* = 31) were obtained from TCGA and GEPIA, respectively. (**A**) Empirical cumulative distribution functions (ECDF) of expression of *MTDH* in cancer (*dot line*) and normal tissues (*solid line*) were calculated. The maximum distance (D${}^{-}$) between the curves was calculated using Kolmogorov–Smirnov test. (**B**) The average fraction of cases (*n* = 10,953) with or without alterations (mutation, fusion, amplification, or deletion of the *MTDH* gene in cancer tissues (*n* = 31) from TCGA. The difference between altered and not altered were tested using *t*-test. (**C**) A scatter plot of the *MTDH* and *RKIP* expression in patient tissue (*n* = 1084) from TCGA, Breast Cancer cohort. Pearson’s correlation coefficient (r) was used to evaluate the correlation between the two genes and test for association.

MTDH is broadly found in the nucleus or the cytoplasm of different types of malignant cells [11]. Its expression is specifically regulated by nuclear factor kappa B (NF-κB), and it is also highly related to tumor progression, such as metastasis and angiogenesis. In addition, the high expression of MTDH is connected to the aggressive metastasis in breast, ovarian, and cervical cancers [8,12]. First, we examined the expression profile of MTDH in cancer tissues (*n* = 31) obtained from human samples in the cancer genome atlas (TCGA), and the corresponding normal tissues from GEPIA [13]. We found that the expression of *MTDH* was significantly higher in tumor tissues compared to control tissues (Figure 1A). More specifically, the percentage of tumor tissues at every expression level was higher than normal tissues, as demonstrated by empirical cumulative distribution functions (ECDF). Since genetic changes exert a great influence on not only gene expression but also gene function, we investigated the genetic alterations (mutation, fusion, amplification, or deletion) of the *MTDH* gene obtained from human subjects (*n* = 10,953) in the same set of the TCGA cancer studies. We found that few cancer tissue samples had any genetic alterations (2% of cases) of the *MTDH* gene (Figure 1B). Our data indicate that MTDH was highly expressed in tumor tissues compared to normal tissues along with a lower frequency of genetic alterations of the *MTDH* gene.

### 2.2. MTDH Knockdown Induces RKIP Expression in Breast Cancer Cell Lines

**Figure 2:**
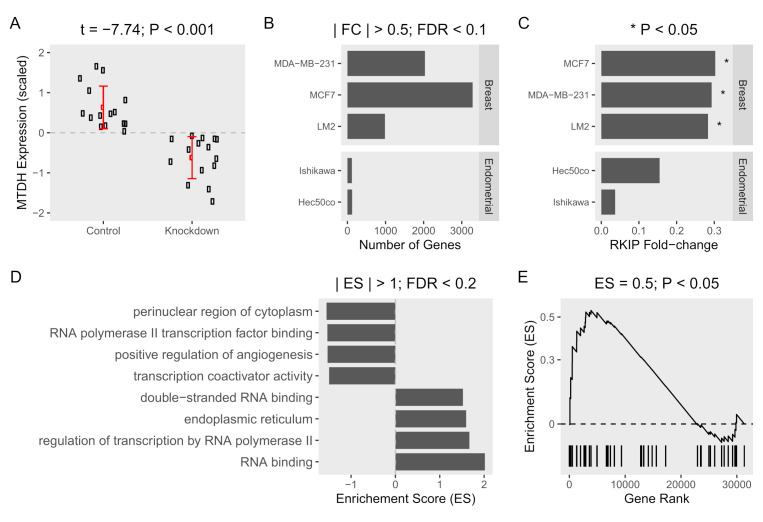
Gene expression and gene set enrichment analysis of *MTDH*-knockdown in cancer cell lines. Datasets obtained from three *MTDH*-knockdown breast cells (MCF-7, MDA-MB-231, and LM2) and two *MTDH*-knockdown endometrial (Ishikawa, and Hec50co) cancer cell lines were compared to control cells (three samples of each cell line). (**A**) Expression of MTDH (scaled fold-change ($log_{2}$)) in five knockdown breast and endometrial cancer cell lines and corresponding five control cells. The mean difference in expression was tested using *t*-test. (**B**) Gene expression was compared between knockdown and control cells in each cell line. Fold-change (FC) was calculated and tested for significance using false-discovery rate (FDR). (**C**) Fold-changes of RKIP in each cell line are shown. (**D**) Genes were ranked based on fold-change from the most positively changed to the most negatively changed in knockdown vs. control cells. Enrichment of MTDH-related gene ontology terms was calculated as the over-representation of the term members in the top or the bottom of the ranked list of differentially expressed genes in one or more breast cancer cell lines. Enrichment scores (ES) > 1 and FDR < 0.2 are shown. (**E**) Enrichment of the “negative regulation of autophagy” term.

In general, RKIP, known as a tumor and metastasis suppressor, is severely downregulated in metastatic breast tissue compared to normal tissue [14]. This is in contrast to MTDH on cancer tissue. To further examine the association of MTDH with RKIP expression in tumor cells, we then performed a systemic analysis using public-access datasets where the *MTDH* gene was knocked down (Table 1). As expected, the relative expression of MTDH in three breast and two endometrial cancer cell lines was lower in the knockdown condition compared to normal control (Figure 2A). When compared to the control cells, ablation of MTDH caused significant fold-changes in expression of many genes. Indeed, we observed that about 1000–3000 genes were differentially expressed in breast cancer cells when the *MTDH* gene was knocked down, but few genes were altered in endometrial cells according to the knockdown of MTDH (Figure 2B). Changes in gene expression were shown more clearly in breast cancer cells than endometrial cells, especially in MCF-7. In the same dataset, RKIP expression was found to be highly affected by the reduction of MTDH. In fact, knockdown of the *MTDH* gene resulted in a 0.3-fold increase in RKIP expression (*p*-value < 0.05) in the three *MTDH*-knockdown breast cells compared to controls (Figure 2C). Additionally, we found an inverse correlation of *MTDH* and *RKIP* expression (Figure 1C) in patient breast cancer cohort from TCGA. Due to the converse expressions of RKIP and MTDH in our data, it is important to determine the molecular mechanism of the correlation between the two proteins in order to understand the regulation of cancer progression.

Besides, we examined the enrichment of MTDH-related gene ontology terms among differentially expressed genes in *MTDH*-knockdown vs. control cells. Generally, the elevated level of MTDH in cancer cells is considered a hallmark for the severity of tumor progression. Several biological functions related to MTDH were enriched in the *MTDH* knockdown (Figure 2D). A positive enrichment score means that the gene numbers at a given term are over-represented in the upregulated genes as a result of MTDH knockdown. The reverse is true for negative enrichment scores and downregulated genes. As expected, gene products in terms related to tumor progression such as positive regulation of angiogenesis were dysregulated in the absence of MTDH. RNA polymerase II transcription factor binding and transcription coactivator binding terms were over-represented between the two experimental groups. Besides, genes in regulation of transcription by RNA polymerase II term were downregulated by *MTDH* knockdown. This strongly indicates a role for MTDH in regulating gene transcription and expression. We additionally found that *MTDH*-knockdown induced significant changes in enrichment of the “negative regulation of autophagy” gene term in the same datasets (Figure 2E). Interestingly, RKIP was suggested to be a negative regulator of autophagy process in our recent study [15]. Taken together, our analysis suggests that the expression of MTDH and RKIP are conversely correlated, and that the *MTDH* gene is functionally related to expression of many genes including *RKIP* in different oncogenic pathways.

### 2.3. MTDH Regulates the Transcription of the *RKIP* Gene

**Figure 3:**
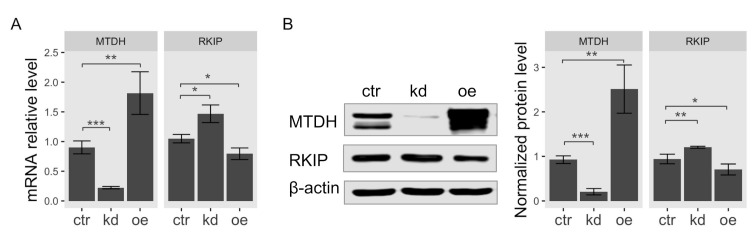
Transcriptional activation of *RKIP* gene in *MTDH*-knockdown or -overexpressing cells. Lentiviral vector with *shMTDH* sequence and FLAG-MTDH plasmid were used for generating knockdown and overexpression in MCF-7 cells, respectively. (**A**) qRT-PCR analysis. The relative mRNA transcripts of MTDH or RKIP in *MTDH*-knockdown cells (*kd*), *MTDH*-overexpressing cells (*oe*), or control cells (*ctr*) were determined by qPCR analysis. The relative levels were normalized to the level of glyceraldehyde 3-phosphate dehydrogenase (GAPDH). Data represent the means ± S.D of the PCR reactions in triplicate. (**B**) Western blot analysis for RKIP expression. Total cellular proteins extracted from either *MTDH*-knockdown cells (*kd*) or *MTDH*-overexpressing cells (*oe*) were subjected to Western blotting analysis (*left*) using antibodies against MTDH or RKIP. The relative expression of each protein normalized to β-actin (an internal control). Data indicate the mean value ± S.D of at least three independent experiments. *** <0.005,** <0.01, * <0.05 *p*-value.

According to the result of gene expression analysis in Figure 1, the observed expression level of RKIP was the most significantly upregulated in breast cancer cell line MCF-7 among *MTDH*-knockdown cells. Therefore, we used MCF-7 cells for further investigation in this study. To determine the functional association of MTDH with *RKIP*, we examined both mRNA and protein levels in human breast cancer MCF-7 cells when the *MTDH* gene was knocked down or overexpressed. First, we examined total *RKIP* mRNA levels in *MTDH*-knockdown and *MTDH*-overexpressing MCF-7 cancer cells using quantitative RT-PCR analysis. Total RKIP transcripts significantly increased when the *MTDH* gene was knocked down but reversely decreased in the overexpression of MTDH (Figure 3A). To verify the interaction between MTDH and RKIP indicated by the results of qRT-PCR and microarrays analyses, we examined RKIP protein levels by Western blotting. We found that the protein level of RKIP was specifically regulated by modulating MTDH expression (Figure 3B). Knockdown of MTDH resulted in a significant increase of RKIP protein level compared to control. By contrast, MTDH overexpression relatively decreased the level of RKIP protein, indicating that the intracellular level of RKIP is somehow regulated by MTDH. Collectively, we suggest that MTDH could be a transcriptional repressor of the *RKIP* gene.

### 2.4. MTDH Binds to the *RKIP* Promoter

**Figure 4:**
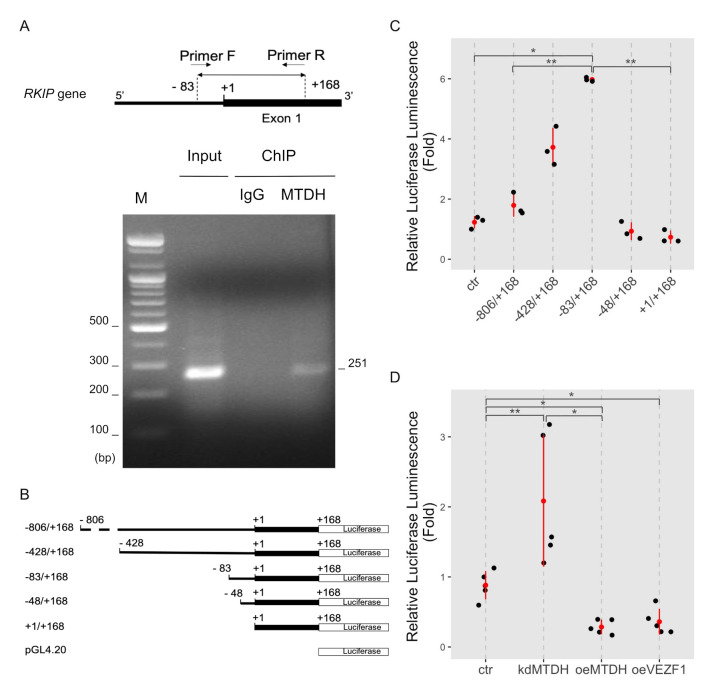
MTDH binding to the *RKIP* promoter. (**A**) Chromatin immunoprecipitation (ChIP) assay. MCF-7 cells were double cross-linked with DSG and 4% formaldehyde and lysed with sonication. DNA fragments were immunoprecipitated with anti-MTDH antibodies (MTDH) or IgG as a negative control (IgG). Specific DNA fragments were amplified with a pair of primers. For input control, either none or purified DNA fragments were amplified. PCR primers used for ChIP assay were created to specifically amplify a 256 bp-DNA fragment (from −83 to +168 on the upstream region of *RKIP* gene, as shown in the top of (A). Amplified DNA fragments were separated on a 1% agarose gel and visualized by EtBr staining. The first lane indicates 100 bp size markers. (**B**) A schematic illustration of the constructs for the luciferase *RKIP* promoter assay. The serially deleted *RKIP* promoter DNA fragments (−806/+168, −428/+168, −83/+169, −48/+168, and +/+168) were inserted into the pGL4.20 luciferase vector. (**C**) The *RKIP* promoter activity. The relative luciferase activities were determined in MCF-7 cells transfected with serially deleted constructs of the *RKIP* promoter. Data represent fold-changes from three independent experiments. ** <0.01, * <0.05 *p*-value. (**D**) *RKIP* promoter activity upon modulating MTDH expression. MCF-7 cells were transfected by none (*ctr*), *shMTDH* (*kdMTDH*), *FLAG-MTDH* (*oeMTDH*), or *VEZF1* plasmids (*oeVEZF1*) and used in luciferase assay with the *RKIP* promoter −83/+168 construct. Data represent fold changes from five independent experiments. ** <0.01, * <0.05 *p*-value.

We determined whether MTDH binds to the RKIP promoter using the chromatin immunoprecipitation (ChIP) analysis. Since MTDH contains no DNA-binding domain as previously described [10], we used a dual cross-linking ChIP method that allows effective transcription co-factors linking to DNA. The targeted regions on the *RKIP* promoter (251 bp, from −83 to +168) for ChIP analysis are represented in Figure 4A. The ChIP assay using anti-MTDH antibodies showed that MTDH was physically linked to the *RKIP* promoter region, but not in the case of using negative IgG (Figure 4A). Interestingly, when we used the conventional ChIP assay by a single cross-linking DNA-protein step with 4% formaldehyde, we were unable to detect any association of MTDH with the *RKIP* promoter (Figure S1). These results suggest that MTDH could be a transcriptional repressor that inhibits the transcription of *RKIP*. This repression could occur via an indirect association with other transcription factors that bind to a nearby region of the RKIP transcription start site.

To further confirm these results, we performed the luciferase reporter assay using the *RKIP* promoter. First, we constructed reporter vectors containing serial deletions of the *RKIP* promoter DNA (−806 to +168; −428 to +168; −83 to +168; −48 to +168; and +1 to +168) linked to the *firefly* luciferase gene (pGL4.20 vector), as shown in (Figure 4B). Then, we tested the *RKIP* promoter activity using these constructs in MCF-7 cells. Interestingly, deleting the upstream region of *RKIP* promoter up to −83 caused a gradual increase in the promoter activity. The promoter region of −83/+168 was the most active. However, further deletions of the *RKIP* promoter (either −48/+168 or +1/+168) led to a significant decrease in the promoter activity (Figure 4C). Similar results were observed by Zhang et al. [16]. We further discuss the possible regulatory mechanism in the Discussion Section.

To test how MTDH influences the *RKIP* promoter activity, we measured the luciferase activity upon perturbing MTDH expression (either knockdown or overexpression). The promoter construct −83/+168 showed the highest activity in the assay. As expected, the *RKIP* promoter activity highly increased in *MTDH*-knockdown MCF-7 cells compared to control cells. Conversely, the promoter activity dramatically decreased in *MTDH*-overexpressing MCF-7 cells (Figure 4D). In addition to MTDH-dependent regulation of RKIP expression, the overexpression of vascular endothelial zinc finger 1 (VEZF1), a transcription factor, slightly inhibited the *RKIP* promoter activity. VEZF1 has been suggested as a functional partner of RKIP according to our previous study [17]. Accordingly, these results indicate that MTDH plays an important role in the transcriptional regulation of *RKIP* gene through either direct or indirect binding with other factors to the *RKIP* promoter region.

### 2.5. Functional Association between MTDH and VEZF1 during Transcriptional Activation of *RKIP* Gene

**Figure 5:**
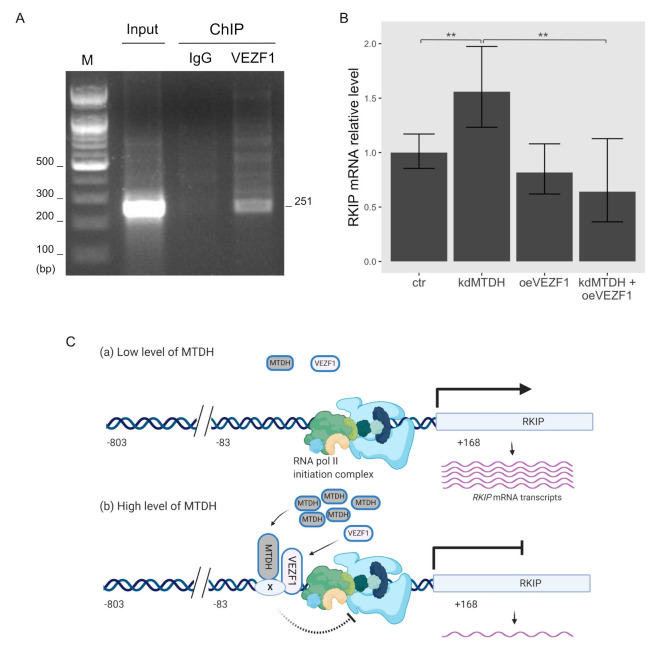
Functional linkage between VEZF1 and MTDH in *RKIP* transcription. (**A**) Chromatin immunoprecipitation (ChIP) assay of the *RKIP* promoter with VEZF1. MCF-7 cells were cross-linked with 4% formaldehyde and lysed with sonication. DNA fragments were immunoprecipitated with anti-VEZF1 antibodies (VEZF1) or IgG as a negative control (IgG), and a specific DNA fragment of 251-bp was amplified with a pair of *RKIP* primers described in “Materials and Methods”. For input control, the purified DNA fragments were amplified. Amplified DNA fragments were separated on a 1% agarose gel and visualized by EtBr staining. The first lane indicates 100 bp size markers. (**B**) qRT-PCR analysis. The relative RKIP mRNA transcripts were determined in each condition: control (*ctr*), MTDH knockdown (*kdMTDH*), VEZF1 overexpression (*kdVEZF1*), and MTDH knockdown and VEZF1 overexpression (*kdMTDH + oeVEZF1*). PCR products amplified by RKIP primers (Figure 3A) were normalized to GAPDH. Data represents the means ± S.D of the PCR reactions in triplicate. ** <0.01 *p*-value (**C**) A schematic diagram for the MTDH-dependent inhibition of RKIP transcription. The transcriptional activity of RKIP gene is elevated in low level of MTDH (a), and suppressed in high level of MTDH (b), presumably by blocking the action of RNA polymerase II initiation complex. Factor X indicates an unknown protein. This figure was created with BioRender.com, accessed on 11 February 2021.

As mentioned above, MTDH could not directly bind to the *RKIP* promoter region. Instead, it might require other transcription factors to regulate *RKIP* gene. According to our recent work using weighted gene co-expression network analysis (WGCNA), RKIP transcripts were functionally associated with the transcription factor VEZF1. In particular, their expression is inversely correlated with each other [17], which is a similar pattern to the RKIP and MTDH association shown above (Figure 2 and Figure 3). Thus, MTDH-dependent transcriptional inhibition of the *RKIP* gene could require the functional connection with VEZF1. To test this possible mechanism, we first examined the binding ability of VEZF1 to the *RKIP* promoter region using ChIP assay. As expected, the VEZF1 protein bound to the *RKIP* promoter (Figure 5A), and the overexpression of VEZF1 strongly inhibited the transcriptional activity of the *RKIP* gene in the luciferase reporter assay. The activity of the −83/+168 *RKIP* promoter construct (last bar in Figure 4D) was very similar to the transcriptional activity exhibited in cells overexpressing *MTDH*. These data suggest that the transcription factor VEZF1 and MTDH could form a DNA-binding complex and subsequently repress *RKIP* gene expression in cancer cells.

We further examined how the two proteins MTDH and VEZF1 influence the transcriptional activation of *RKIP* gene using quantitative PCR analysis in cells in which the expression of these proteins was perturbed. As expected, knocking down *MTDH* increased the transcriptional activation of *RKIP* gene, and overexpressing VEZF1 significantly decreased the *RKIP* mRNA transcripts (Figure 5B). However, in the case of the double perturbation (*MTDH*-knockdown and *VEZF1*-overexpression), the amount of RKIP transcripts was similar to the value obtained from *VEZF1*-overexpressing cells. This result indicates that MTDH-dependent inhibition of RKIP expression is not observed at the high level of VEZF1 protein.

## 3. Discussion

Several previous studies suggest that MTDH could contribute to cancer progression, and it is considered a hallmark protein of metastatic cancers. Indeed, MTDH stimulates EMT and cancer metastasis, and it also exhibits chemoresistance to cancer drugs. Furthermore, MTDH stimulates proliferation of cancer cells [18,19]. The high level of this protein in cancer tissues also correlates with poor prognosis [19,20]. In this study, we investigated the molecular mechanism of how MTDH directs and stimulates cancer progression, and we found that the elevated level of MTDH in cancer cells suppress the transcription of the *RKIP* gene. Additionally, the abundance mRNA transcripts of *MTDH* inversely correlated (*r* = −0.15; *p*-value < 0.001) with the *RKIP* gene according to a systemic analysis of breast tissue samples in The Cancer Genome Atlas (TCGA) data. Based on these results, we suggest that understanding the mechanism underlying the regulatory relationship between these two proteins could provide some key solutions for developing new cancer therapy. MTDH, also known as AEG-1, can act as a transcriptional co-factor in cancer signals. It directly interacts with the p65 subunit of NF-κB and facilitates the transcription of NF-κB downstream genes necessary for the cancer invasion process [21,22]. In addition, MTDH can physically bind to the staphylococcal nuclease domain-containing protein1 (SND1), which acts as a nuclease of RNA-induced silencing complex (RISC). Indeed, binding of MTDH and SND1 stimulates the RISC activity and consequently facilitates the gene silencing of tumor suppressor mRNAs via either small interfering RNA (siRNA) or microRNA (miRNA) processes [23,24,25]. Besides, SND1 is a co-factor that can interact with general transcriptional machinery such as TFIIB, TFIIH, and TFIIE [26]. Therefore, MTDH could regulate the expression of tumor-associated genes, including *RKIP* by cooperating with other transcription factors.

Low expression of RKIP, known as an anti-metastatic factor, is often reported in aggressive breast cancer tissues [14,27]. Furthermore, downregulated RKIP in cancer cells is highly associated with the early step of cancer progression. Indeed, decreased RKIP in cancer cells promotes the epithelial-to-mesenchymal transition (EMT), which is an essential preceding process for cancer metastasis [14]. Based on the known functions of MTDH and RKIP and our results, the two proteins could be functionally connected during cancer progression. We found that RKIP expression was inversely linked to the intracellular level of MTDH in cancer cells (Figure 2). Again, we suggest that MTDH could be a functional adaptor that interacts with other transcriptional factors or a mediator for the activity of oncogenic proteins.

According to our previous studies, RKIP showed functional connections with many other autophagy-related proteins and, in fact, it inhibited autophagy in cells under the limited nutrients [15,17]. Autophagy is a self-eating catabolic process that supplies nutrients necessary for the survival of cancer cells that need more energy during cancer progression. Because autophagy is sometimes beneficial for cancer survival, RKIP-dependent inhibition of autophagy could be a possible mechanism for anti-EMT or anti-metastasis. Therefore, the regulation of RKIP expression is a target for cancer therapy. Gene-set enrichment analysis showed that MTDH interacts with several gene products related to autophagy, apoptosis, and angiogenesis. In particular, the reduction of MTDH strongly upregulated the expression of RKIP especially in breast cancer cells (Figure 2).

MTDH regulates multiple genes at the transcriptional level. It directly interacts with p65 subunit of NF-κB as a transcription co-factor [21]. Additionally, it regulates the transcriptional activity of some oncogenic genes. A recent study demonstrated that MTDH is recruited to the Forkhead box M1 (FOXM1) transcriptional complex and acts as a mediator for gene expression [28]. MTDH not only regulated the FOXM1 stability but also enhanced FOXM1-dependent transcription. Here, we suggest that MTDH could be a transcription repressor of the *RKIP* gene. Indeed, MTDH is specifically recruited to the *RKIP* promoter region, specifically the −83/+168 region. Interestingly, the promoter activity of *RKIP* gene gradually increased with serial deletions from the tentative promoter (−806 to +168) and reached the maximum value at the −83/+168 region (Figure 4). This possibly suggests that the upstream of *RKIP* promoter could be involved in interactions with other transcription factors that negatively regulate the expression of RKIP or that the RNA polymerase complex with general transcription factors bound to the −83/+168 region is good enough for the activation of *RKIP* gene. A similar result was also observed in another study by Zhang et al. in 2013 [16]. As a result, the elevated level of MTDH in cancer can lower RKIP protein through the transcriptional repression possibly together with other transcription factors.

Based on our recent study, the expression of *RKIP* gene is functionally connected to the vascular endothelial zinc finger 1 (VEZF1), a transcription factor [17]. Therefore, VEZF1 might also be involved in MTDH-dependent regulation of the gene. In this study, we showed that VEZF1 tightly bound to the region −83 to +168 on the *RKIP* promoter, and it significantly inhibited the promoter activity, similar to MTDH. These observations suggest that direct association of MTDH with VEZF1 or indirect connection with unknown proteins, as shown in Figure 5C, could reduce the transcriptional activity of *RKIP* gene and control its intracellular protein level in cancer. VEZF1 is highly expressed in endothelial cells, and it is an important regulator for angiogenesis in cancer [29,30,31,32]. By contrast, RKIP negatively regulates angiogenesis and inhibits cancer invasion and metastasis [33,34]. Considering these results, VEZF1/MTDH-mediated inverse regulation of the *RKIP* gene could be a critical mechanism in tumorigenesis. However, we need further experiments to show how the two proteins VEZF1 and MTDH cooperatively regulate the expression of *RKIP* gene during cancer progression.

In conclusion, MTDH and RKIP proteins are important prognostic markers in human cancers. They exhibited an inverse correlation in expression in malignant cancer cells. MTDH transcriptionally repressed *RKIP* gene. Thus, perturbing the MTDH–RKIP relation in human cancer might serve as a target developing effective anticancer therapy.

## 4. Materials and Methods

### 4.1. Reagents

Antibodies used in the study were as follows: PEBP1/RKIP (*sc-28837*) and VEZF1 (*sc-365561*) from Santa Cruz Biotechnology (Dallas, TX, USA) and MTDH/LYRIC (*D5Y8R*) and β-actin (A5441) from Sigma Aldrich (St. Louis, MO, USA). Secondary antibodies against rabbit (*STAR208P*) or mouse (*STAR117P*) were purchased from Bio-Rad (Hercules, CA, USA). RPMI-1640 (*11875-119*), fetal bovine serum (FBS; *16000-044*), Dulbecco’s modified Eagle’s medium (DMEM, *11995-065*), Lipofectamine 3000 (*11668-500*), and G418 (*10131-035*) were purchased from Gibco and Life Technologies (Carlsbad, CA, USA). Anti-FLAG M2 affinity gel (*A2220*) and Poly-L-lysine solution (*P8920*) were from from Sigma-Aldrich (St. Louis, MO, USA). M2 lysis buffer (*85111*), protease inhibitor cocktails (*78441*), Enhanced ChemiLuminescence (ECL) detection system (*34080*), and Disuccinimidyl glutarate/DSG (*20593*) were from Thermo Scientific (Waltham, MA, USA). Trizol reagent (*15596026*) was from Invitrogen, Carlsbad, CA, USA. QuantiNova^TM^ SYBR Green RT-PCR Kit (*208154*) was from QIAGEN Inc., Hilden, Germany.

### 4.2. Gene Expression Analysis of *MTDH*-Knockout Cells

Three microarray datasets of *MTDH*-knockdown cells obtained from gene expression omnibus (GEO) using GEOquery (11752295,17496320) are shown in Table 1. Probe intensities of *MTDH*-knockdown cells and control cells were normalized and compared using Limma [35]. Genes were ranked based on fold-change from the most positively changed to the most negatively changed. Enrichment of MTDH-related gene ontology terms was calculated as the over-representation of the term members in the top or the bottom of the ranked list of differentially expressed genes in one or more Breast cancer cell lines using fgsea [36]. Expression of RKIP at the two different conditions was validated using the two-tailed student *t*-test.

**Table 1:** Sources and contacts of datasets.

Tissue	Cell Line	GEO ID	N	Ref.
	MCF7	GSE59055	6	[37]
Breast	MDA-MB-231	GSE59057	6	[37]
	LM2	GSE9187	6	[7]
Endometrium	Ishikawa	GSE27828	6	[38]
	Hec50co	GSE26134	6	[38]

### 4.3. *MTDH* Gene Expression Analysis in Cancer Tissues

Gene expression datasets of human cancer tissues (*n* = 31) were obtained from the cancer genome atlas (https://www.cancer.gov/tcga, accessed on 11 February 2021) and corresponding normal tissue samples from GEPIA (http://gepia.cancer-pku.cn/, accessed on 11 February 2021). Percentages of samples with a given MTDH expression or lower in cancer or normal tissue were calculated as the empirical cumulative distribution function (ECDF). Gene copy numbers alterations (mutation, fusion, amplification, or deletion) of the *MTDH* gene from human subjects (*n* = 10,953) in TCGA were downloaded from cBioPortal (http://cbioportal.org/, accessed on 11 February 2021). Percentages of samples with and without altered *MTDH* gene were calculated as the empirical cumulative distribution function (ECDF).

### 4.4. Dual Cross-Linking Chromatin Immunoprecipitation Assay

MCF-7 cells were treated with disuccinimidyl glutarate (DSG) for capturing protein–protein complexes for 30 min at room temperature (RT) at the 80% confluence. Subsequently, formaldehyde was added drop-wise to the media to a final concentration of 0.75% to cross-link proteins to DNA, and the culture dish was incubated with gentle rotation for 10 min at RT. Then, glycine is added to a final concentration of 125 mM into the media to quench the formaldehyde and terminate the cross-linking reaction for 5 min at RT. The crossed-linked cells were lysed in M2 lysis buffer (50 mM Tris-HCl, pH 7.4, 150 mM NaCl, 1 mM EDTA, 1% Triton X-100, and protease inhibitor cocktails). Cell lysates were sonicated to obtain sheared chromatin DNA at an average fragment size of 200–1000 bp. After sonication, cell debris was removed by centrifugation for 10 min and 4 ${}^{\circ }$C, 8000× *g*, and then the supernatant was transferred to a new tube. The prepared chromatin DNA fragments were used for immunoprecipitation using a specific antibody-conjugated beads with rotation at 4 ${}^{\circ }$C, overnight. Mixtures of beads with nonspecific immunoglobulin (IgG) were used as a negative control. Then, the following washes were implemented: once in low salt wash buffer, once in high salt wash buffer, and once in LiCl wash buffer. After each wash, the supernatant was removed by centrifuge for 1 min at 2000× *g*. The elution buffer (120 μL) was added to the protein A/G beads and vortexed slowly for 15 min at 30 ${}^{\circ }$C. The supernatant was collected by centrifuge for 1 min at 2000× *g*. Finally, the eluted DNA samples were further purified using a PCR purification kit. The eluted DNA and input DNA samples were subjected to PCR amplification for *RKIP* gene using a pair of primers: *5${}^{\prime }$-GTGACGTGGGGCGGTG-3${}^{\prime }$* and *3${}^{\prime }$-TGACATGCAGCGGGTGCT-5${}^{\prime }$*.

### 4.5. Cell Culture and Transfection

MCF-7 cells were cultivated in RPMI-1640 containing 10% FBS and cultured at 37 ${}^{\circ }$C in a humidified atmosphere of 5% CO${}_{2}$. Cells were transfected by plasmids using Lipofectamine 3000 as described by the manufacturer’s instruction (Invitrogen).

### 4.6. Production of Recombinant Lentiviral Particles and Infection

Lentiviral particles were produced using a third-generation lentivirus packaging mix (Applied Biological Materials Inc., Richmond, BC, Canada). In brief, the shRNA-MTDH construct and the lentiviral packaging plasmids were simultaneously transfected into A293T cells. After incubation, the supernatant containing viral particles was collected and stored at −80 ${}^{\circ }$C until used.

### 4.7. Western Blot Analysis

Cells were lysed in a buffer containing 50 mM Tris-Cl (pH 8.0), 150 mM NaCl, 0.1% SDS, 0.5% sodium deoxycholate, 0.02% sodium azide, and protease inhibitor cocktails. The protein concentration of total cell lysates was determined using the BCA method. Total proteins (30 μg) were separated by 10% SDS-PAGE unless indicated otherwise and transferred to a nitrocellulose membrane using a wet transfer system (Bio-Rad) for 90 min at 80 V. The membrane was blocked for 1 h at room temperature in TBST (10 mM Tris, pH 7.5, 150 mM NaCl, and 0.1% Tween 20) with 5% skim milk. After incubation with primary antibodies overnight at 4 ${}^{\circ }$C in TBST with 5% skim milk, the membrane was washed three times in TBST for 10 min each and then incubated with secondary antibodies in TBST for 1 h. The membrane was subsequently washed three times with TBST for 10 min. Proteins were quantified using the NIH ImageJ program (version 1.49). The graphical data represent the mean (±S.D) of at least three independent experiments.

### 4.8. RNA Extraction and RT-qPCR Analysis

Total RNAs were extracted from MCF7 breast cancer cells using Trizol reagent according to the manufacture’s manual. RNA targets were quantified by the real-time one-step RT-PCR analysis using SYBR Green I detection from QuantiNova${}^{\mathrm{TM}}$ SYBR Green RT-PCR kit. GAPDH was used as an internal control. Primers used in the study: RKIP-Forward: 5${}^{\prime }$-GTCACACTTTAGCGGCCTGT-3${}^{\prime }$, RKIP-Reverse: 5${}^{\prime }$-CTCTCCGATTATGTGGGCTC-3${}^{\prime }$; MTDH-Forward: 5${}^{\prime }$-GTAAACGTGATAAGGTGCTGACT-3${}^{\prime }$, MTDH-Reverse: 5${}^{\prime }$-CGGTG GTAACTGTGATGGTATTT-3${}^{\prime }$; GAPDH-Forward: 5${}^{\prime }$-TGCACCACCAACTGCTTAGC-3${}^{\prime }$, GAPDH-Reverse: 5${}^{\prime }$-GGCATGGACTGTGGTCATGAG-3${}^{\prime }$. RT-PCR data were processed based on Delta-Delta CT using “pcr package” [39]. *p* values < 0.05 were considered significant. Other analysis have been done in R [40] (Vienna, Austria).

### 4.9. Luciferase Promoter Assay

Cells were plated at $2\times 10^{5}$ cells/ml in 96-well plates. Promoter-*Firefly* luciferase pLG4.20 constructs were transiently transfected together at a ratio 10:1 with pRL-SV40 (Promega, Madison, WI, USA) vector expressing *Renilla* luciferase into MCF-7 cells using lipofectamine 3000. The luciferase activity was measured by the Dual-Glo Luciferase assay system as described in the manufacture’s manual (Promega). After 48 h, an equal volume of Dual-Glo reagent to the volume of culture medium was added into each well of the plate. The *Firefly* luminescence was measured after incubation for 10 min at 20–25 ${}^{\circ }$C using GloMax Explorer (Promega). Subsequently, the *Renilla* luminescence was measured by incubating the wells with Dual-Glo Stop and Glo Reagent at 20–25 ${}^{\circ }$C for 10 min. The ratio of *Firefly*: *Renilla* luminescence in each well was calculated. The values were normalized to the ratio obtained from a control well.

### 4.10. Statistical Analysis

Each experiment was independently conducted at least three times, and the data were expressed as the mean value (±S.D). Statistical significance between two groups was determined by Student *t*-test using the Prism software (GraphPad Prism, La Jolla, CA, USA) and R [40] (Vienna, Austria). *p* values < 0.05 were considered significant.

## Data Availability

Not applicable.

## Supplementary Materials

The following are available at https://www.mdpi.com/1422-0067/22/6/3052/s1.
