# Supplementary figures and images for: Transcriptional Repression of Raf Kinase Inhibitory Protein Gene by Metadherin during Cancer Progression
Source: Int J Mol Sci. 2021 Mar 17;22(6):3052. doi: 10.3390/ijms22063052 (PMC8002422; doi:10.3390/ijms22063052)

Input

ChIP

IgG

MTDH abs

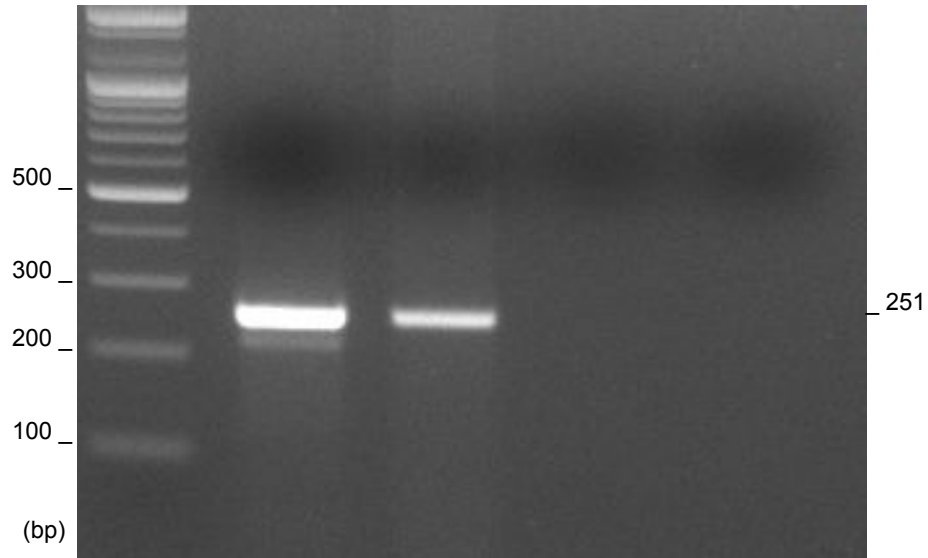

Supplement: Supplementary file 1 [file ijms-22-03052-s001.pdf]
